# Isolation of the Novel Strain *Bacillus amyloliquefaciens* F9 and Identification of Lipopeptide Extract Components Responsible for Activity against *Xanthomonas citri* subsp. *citri*

**DOI:** 10.3390/plants11030457

**Published:** 2022-02-07

**Authors:** Xin Wang, Liqiong Liang, Hang Shao, Xiaoxin Ye, Xiaobei Yang, Xiaoyun Chen, Yu Shi, Lianhui Zhang, Linghui Xu, Junxia Wang

**Affiliations:** 1 Integrative Microbiology Research Centre, College of Plant Protection, South China Agricultural University, Guangzhou 510642, China; xinwang@stu.scau.edu.cn (X.W.); liangliqiong808@163.com (L.L.); shaohang126@163.com (H.S.); yxx13413646894@163.com (X.Y.); belinda0213@163.com (X.Y.); cxy18320721873@163.com (X.C.); Yushi201503@163.com (Y.S.); lhzhang01@scau.edu.cn (L.Z.); 2 Guangdong Province Key Laboratory of Microbial Signals and Disease Control, South China Agricultural University, Guangzhou 510642, China

**Keywords:** citrus canker, *Xanthomonas citri* subsp. *citri*, antagonistic bacteria, biological control, *Bacillus amyloliquefaciens*, lipopeptides

## Abstract

Citrus canker, caused by *Xanthomonas citri* subsp. *citri* (*Xcc*), is a quarantine disease that seriously affects citrus production worldwide. The use of microorganisms and their products for biological control has been proven to be effective in controlling *Xanthomonas* disease. In this study, a novel *Xcc* antagonistic strain was isolated and identified as *Bacillus amyloliquefaciens* F9 by morphological and molecular analysis. The lipopeptide extract of *B. amyloliquefaciens* F9 (F9LE) effectively inhibited the growth of *Xcc* in an agar diffusion assay and restrained the occurrence of canker lesions in a pathogenicity test under greenhouse conditions. Consistent with these findings, F9LE treatment significantly inhibited the production of extracellular enzymes in *Xcc* cells and induced cell wall damage, with leakage of bacterial contents revealed by scanning electron microscopy and transmission electron microscopy analyses. In addition, F9LE also showed strong antagonistic activity against a wide spectrum of plant pathogenic bacteria and fungi. Furthermore, using electrospray ionization mass spectrometry analysis, the main antimicrobial compounds of strain F9 were identified as three kinds of lipopeptides, including homologues of surfactin, fengycin, and iturin. Taken together, our results show that *B. amyloliquefaciens* F9 and its lipopeptide components have the potential to be used as biocontrol agents against *Xcc*, and other plant pathogenic bacteria and fungi.

## 1. Introduction 

Citrus canker is a serious bacterial citrus disease that causes significant economic losses in citrus-producing areas worldwide [1]. This disease is caused by the Gram-negative bacterium *Xanthomonas citri* subsp. *citri* (*Xcc*). Citrus canker is highly contagious and spreads rapidly, and is extremely harmful to the citrus industry [2]. Most commercial citrus varieties are susceptible hosts for *Xcc* infection [3,4]. For many years, the most common approach to controlling citrus canker has been to eradicate symptomatic citrus trees to prevent the spread of the pathogens. Spraying of chemical bactericides such as copper and antibiotics is widely applied to prevent and treat *Xcc* infections [5,6,7]. However, the excessive and frequent use of these chemical pesticides has caused environmental pollution, pesticide residues and drug resistance [8]. Therefore, there is an urgent need to search for new alternatives to develop safe, nontoxic and environmentally friendly bactericides to control this disease. In recent years, biological control—i.e., the use of biocontrol agents or their biologically active metabolites—has attracted much attention [5,9,10,11,12]. 

Microorganisms that are used as biocontrol agents have the characteristics of high specificity against target pathogens and low mass production cost [13]. *Bacillus* is currently the most studied antagonistic microorganism, and there are numerous reports about its inhibitory effect on a variety of phytopathogens [5,10,14,15,16,17,18], induction of plant system resistance and improvement of plant response to different stresses [19,20]. The feature contributing to its success is its capacity to produce many biologically active metabolites, especially the nonribosomal synthetic cyclic lipopeptides surfactin, iturin and fengycin. For instance, surfactin produced by *B. amyloliquefaciens* KPS46 is required for biocontrol against *X. axonopodis* pv. *glycines* [21]. Iturin-like lipopeptides are essential components in the biocontrol library of *B. subtilis* against the cucurbit pathogenic bacterium *X. campestris* pv. *cucurbitae* [17]. Surfactins produced by *B. velezensis* 9D-6 were found to inhibit the in vitro growth of bacterial (such as *Ralstonia solanacearum*, *X. campestris* and *X. euvesicatoria*) and fungal (such as *Alternaria solani*, *Cochliobolus carbonum* and *F. oxysporum*) pathogens [22]. In particular, different *Bacillus* spp. have shown biocontrol effects on *Xcc* such as in vitro inhibition of growth [23] or by lysis of bacterial cells [5,24]. The application of *B. subtilis* WG6-14 and TKS-1 24 h prior to *Xcc* inoculation was able to reduce citrus canker development in *Citrus aurantifolia* [25]. A significant reduction in citrus canker growth under field and greenhouse conditions was obtained by single-spray pre-inoculation of *B. subtilis* in *C. aurantifolia* [26,27]. Furthermore, inoculation with *B. subtilis* LE24, *B. tequilensis* PO80 or *B. amyloliquefaciens* LE109 or their crude lipopeptides 1 day after *Xcc* infection significantly reduced citrus canker development [28]. In view of the above, the aims of this study were to isolate and identify a novel *B. amyloliquefaciens* strain from a local farm in southern China against the southern China pathogen *Xcc* jx-6 under both laboratory and greenhouse conditions, isolate and identify the antimicrobial compounds produced by the strain, and determine the compounds’ function in biological processes related to bacterial virulence. The findings in this work form a basis for developing promising strategies for the control of the plant pathogen *Xcc*.

## 2. Results

### 2.1. Isolation and Screening of Antagonistic Bacteria of Xcc 

**Figure 1:**
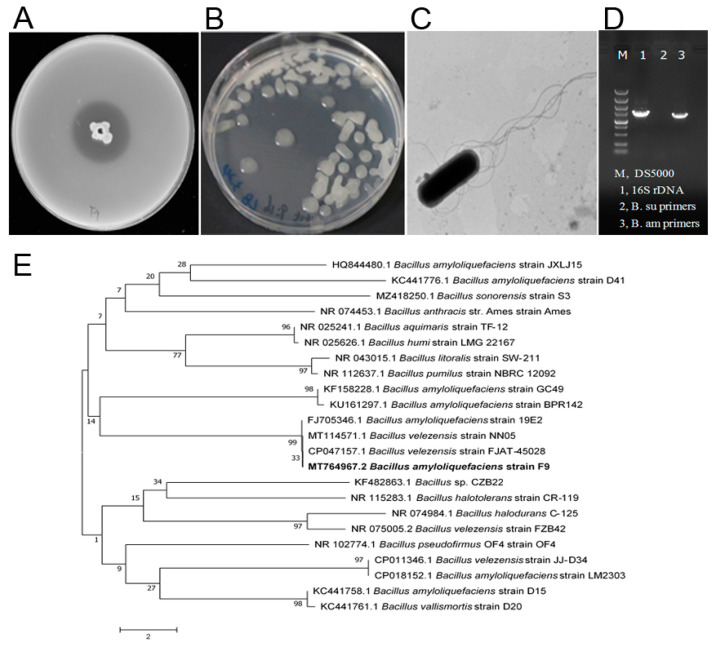
Isolation and characterization of a new antagonistic strain, F9, against *X. citri* subsp. *citri.* (**A**) Agar diffusion assay. The strain F9 showed an antagonistic effect against *Xcc* jx-6. (**B**) Colonies of antagonistic strain F9 grown on LB agar plates at 30 °C for 24 h. (**C**) The cells of strain F9 were observed by transmission electron microscopy. (**D**) Agarose gel electrophoresis analysis of the β-mannanase gene of *Bacillus subtilis* and *Bacillus amyloliquefaciens*. (**E**) Analysis of the phylogenetic tree of strain F9 based on 16S rDNA gene sequences and related bacteria with the neighbor-joining method with a bootstrap value of 1000 replicates. The bar indicates an estimated sequence divergence of 2%.

To isolate antagonistic strains against *Xcc*, ten rhizosphere soil samples were collected from different healthy citrus orchards of Jiangyong County, Hunan Province, China. On LB agar plates containing the pathogen *Xcc* jx-6 as an indicator, the dual culture method was used to isolate and screen the microorganisms that inhibit the growth of *Xcc*. From our collected soil samples, 22 isolates were screened and identified as having antibacterial activity against strain *Xcc* jx-6. These strains displayed inhibition zones ranging from 10 to 30 mm in diameter in an agar diffusion assay (Supplementary Table S1). Among them, the strain F9 with its culture supernatant showed strong antibacterial activity against *Xcc* jx-6, with its inhibitory zone being about 26.5 ± 0.2 mm in diameter (Figure 1A), so it was selected for further study.

### 2.2. Characterization of a New Antagonistic Strain Bacillus amyloliquefaciens F9 against Xcc

In order to obtain a more discriminated classification of the strain F9 at the species level, various experiments, including observation of its cellular morphology, characterization of its biochemical and physiological properties, homologous analysis of its 16S rDNA sequences and specific analysis of its β-mannanase gene, were conducted as described in the Materials and Methods section. The colonies of antagonistic strain F9 grown on LB agar plates at 30 °C for 24 h were white, centrally convex, translucently circular and with irregular margins, and became dried and wrinkled with the prolongation of culture time (Figure 1B). Cells of strain F9 were observed to be typically rod-shaped under scanning electronic microscopy, with blunt ends and peritrichous flagella (Figure 1C). The strain F9 grows at pH 6.0–8.0 (optimum pH 7.0) in anaerobic culture and in the presence of 2–7% (*w*/*v*) NaCl. Catalase, citrate utilization, acid production from glucose and hydrolysis of starch, casein and gelatin were all positive. Indole production, tyrosine hydrolysis and enzyme activities of oxidase, urease, phenylalanine deaminase and lecithin enzymes were all negative (Table 1). These phenotypic properties indicate that the strain F9 belongs to the genus *Bacillus*. 

At the molecular level, the partial 16S rDNA sequence (1455 bp) was registered in GenBank with the accession number MT764967.2 and shares 99% homology with the corresponding *B*. *amyloliquefaciens* sequence. The phylogenetic analysis revealed that strain F9 has the closest genetic relationship with *B. amyloliquefaciens* strain 19E2 (Figure 1E). In addition, a specific DNA fragment (about 1500 bp) was amplified from strain F9 by primer pairs (Bam-man-1F and Bam-man-1R) specific for the β-mannanase gene of *B. amyloliquefaciens* (Figure 1D, lane 3), but not by primer pairs (Bsu-man-1F and Bsu-man-1R) specific for *Bacillus subtilis.* These results further confirm that strain F9 belongs to *B. amyloliquefaciens*.

Taken together, the results show that the strain F9 was identified as *B. amyloliquefaciens* and is named as *B. amyloliquefaciens* F9 here.

### 2.3. Biochemical Characteristics of Strain F9 Lipopeptide Extract (F9LE)

*Bacillus* secretes a wide array of bioactive metabolites to antagonize the growth of other microorganisms for survival in a constantly changing environment [10]. Based on observation of the aseptic supernatant of strain F9 with bacteriostatic activity against the *Xcc* jx-6 strain, we speculated that proteins from the initial extract may be the putative antibacterial metabolites of *B. amyloliquefaciens* F9. To this end, the agar diffusion assay was used to test the antibacterial stability of the extract precipitated using ammonium sulfate, which clearly demonstrated resistance to high temperatures of up to 100 °C for 30 min and degradation by proteinase K (Supplementary Table S2). These findings suggest that lipopeptides may be the active components responsible for the antibacterial activity. To confirm this hypothesis, a crude lipopeptide extract from strain F9 was obtained by acetone extraction combined with HCl precipitation and subjected to an antibacterial stability assay. As expected, the antibacterial activity of the acetone extract showed a clear resistance to high temperatures (45.9–100 °C) and degradation by protein K (Table 2). These observations indicate that the lipopeptide extract contains the main compounds with the antimicrobial activity of strain F9. 

### 2.4. Biological Control Efficiency of F9LE on Xcc jx-6 under Greenhouse Conditions

**Figure 2:**
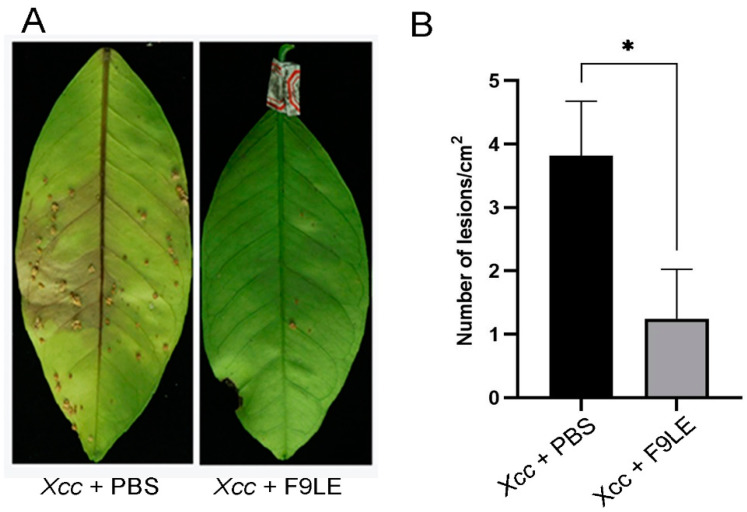
Biological control efficiency of F9 lipopeptide extract (F9LE) on *Xcc* jx-6 under greenhouse conditions. (**A**) The method of spray inoculation was used to control citrus canker in pots. The same amount of *Xcc* jx-6 suspension was sprayed on citrus leaves in advance, bagged after air drying and sprayed with 244 µg/mL F9LE or control PBS after 24 h. (**B**) Quantification of canker lesions in citrus leaves. Mean values were analyzed and separated by Tukey’s HSD test at * *p* < 0.05 after one-way ANOVA. Error bars represent standard deviation.

To evaluate the preventive effect of F9LE on the occurrence and development of citrus canker, the pathogenicity of *Xcc* to the host plant Hongjiang sweet orange was tested by foliar spray inoculation under greenhouse conditions. Twenty-four hours after the *Xcc* jx-6 strain was sprayed on the back of the leaves, the pathogen-infected leaves were sprayed with 244 µg/mL F9LE solution or PBS buffer (set as nontreated samples) as a negative control. These virulence test results show that the negative control PBS-treated leaves displayed serious symptoms with brown canker lesions 30 days after inoculation with *Xcc* jx-6, while the F9LE-treated leaves showed a significantly reduced occurrence of brown canker lesions (Figure 2). These results indicate that F9LE could effectively hinder pathogen *Xcc* infection of sweet orange leaves.

### 2.5. The Effect of F9LE on the Morphology and Ultrastructure of Xcc jx-6 Cells 

**Figure 3:**
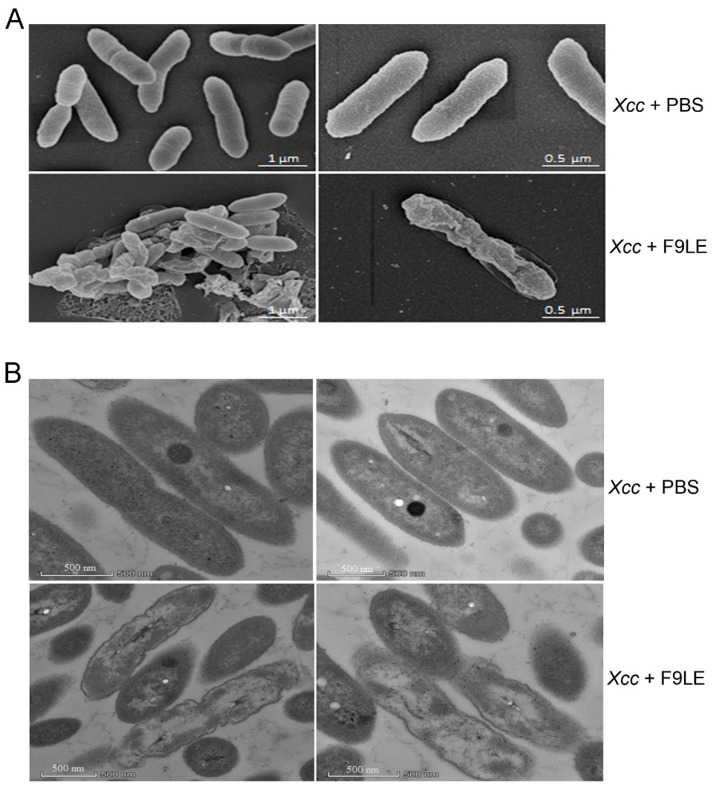
Photographs showing morphological and ultrastructural changes caused by bacteriostatic lipopeptide extract by scanning electron microscopy and transmission electron microscopy. (**A**) The morphological changes of *Xcc* jx-6 cells after exposure to F9LE (200 µg/mL) for 12 h were observed by SEM. (**B**) The ultrastructural changes of *Xcc* jx-6 cells treated with F9LE (200 µg/mL) for 12 h were observed by TEM.

Scanning electron microscopy (SEM) and transmission electron microscopy (TEM) were used to observe the effect of F9LE on the ultrastructure of *Xcc* jx-6 cells. In the SEM study, untreated cells appeared as intact, plump and typically rod-shaped cells with a smooth and bright surface and were dispersed in a unicellular form without any apparent cellular debris. However, the cells treated with 200 µg/mL of the F9LE for 12 h appeared to aggregate, deform and shrink (Figure 3A). The observation of cell aggregation indicates that F9LE can promote biofilm formation.

In the TEM study, the untreated *Xcc* jx-6 cells showed a unified structure with obviously complete envelopes and a cytoplasm comprising uniformly distributed electron-dense structures. After exposure to F9LE at 200 µg/mL for 12 h, many cells displayed a noticeably irregular cell morphology with a clearly thinner cell wall and a highly uneven electron-dense cytoplasm, and some cells even appeared to show cell wall damage and intracellular content leakage (Figure 3B). Therefore, through SEM and TEM analyses, we conclude that F9LE treatment leads to cell wall damage and leakage of the intracellular bacterial content and promotes biofilm formation of *Xcc jx-6* cells. 

### 2.6. Inhibitory Effect of F9LE on Extracellular Enzyme Activity of Xcc jx-6

**Figure 4:**
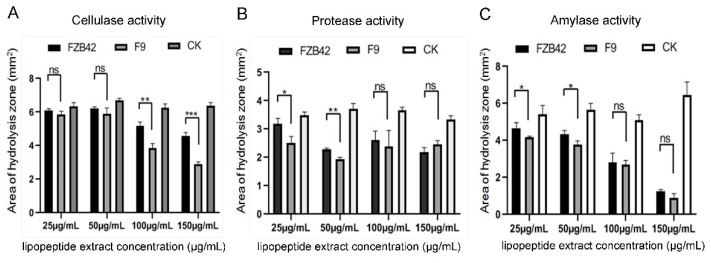
The effect of strain F9 and FZB42 antimicrobial lipopeptides on extracellular enzyme activity of *Xcc* jx-6. (**A**) The cellulase activity assay. (**B**) The protease activity assay. (**C**) The amylase activity assay. The effect of extracellular enzyme activity was qualitatively determined by means of a perforated dot plate. The area of the hydrolysis zone was measured using ImageJ. Error bars represent standard deviation. Mean values were analyzed and separated by Tukey’s HSD test at * *p* < 0.05, ** *p* < 0.01, *** *p* < 0.001, ns: no significance, after one-way ANOVA. Error bars represent standard deviation.

To evaluate whether F9LE has an effect on the virulence factor production of *Xcc* jx-6, the activities of cellulase, protease and amylase were measured using radial diffusion assays. The lipopeptide extract from *B. amyloliquefaciens* FZB42 (FZB42LE) was used as a control here. *Xcc* jx-6 cells mixed with F9LE or FZB42LE at a series of increasing concentrations ranging from 25 to 150 µg/mL were grown on NYG agar plates containing carboxymethylcellulose (for cellulase), skimmed milk (for protease) or starch (for amylase). Based on the calculation of the clearance area of the hydrolysis zone (excluding the area of the colony), it was determined that F9LE has an inhibitory effect on the extracellular enzyme production of *Xcc* jx-6. As shown in Figure 4, both F9LE and FZB42LE treatments clearly reduced the secretion of those three extracellular enzymes of *Xcc* as revealed by the obviously smaller hydrolysis zone areas. Notably, the cellulase activities in samples treated with F9LE (100 and 150 µg/mL) were significantly lower than those in the FZB42LE-treated samples (Figure 4A). F9LE (25 µg/mL) had a similarly stronger inhibitory effect on protease and amylase activities compared with the same concentrations of FZB42LE (Figure 4B,C). Together, these observations suggest that F9LE has a stronger inhibitory effect than FZB42LE on the extracellular enzyme production of the pathogenic strain *Xcc* jx-6.

### 2.7. Determination of Antimicrobial Spectrum of F9LE

**Figure 5:**
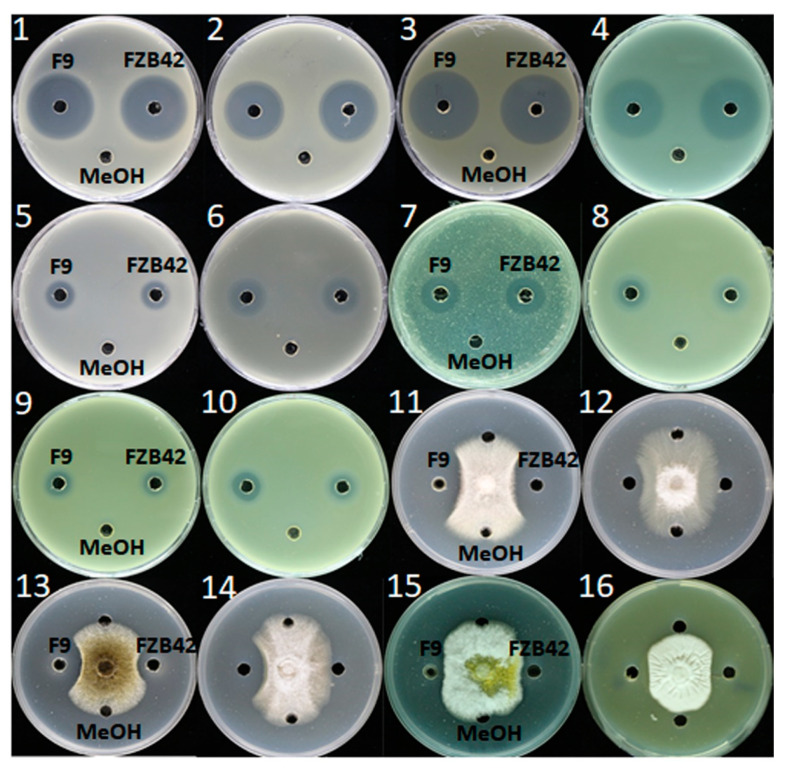
Demonstration of the inhibitory effects of F9LE against 16 phytopathogenic bacteria and fungi (panels 1–16). (1) *X. citri* subsp. *citri* strain jx-6, (2) *X. campestris* pv. *campestris* strain XC1, (3) *X. oryzae* pv. *oryzicola* GDIV, (4) *Ralstonia solanacearum* EP1, (5) *Burkholderia cenocepacia* H111, (6) *Dickeya zeae* EC1, (7) *Pectobacterium carotovorum* Er, (8) *Pantoea ananatis* SC7, (9) *Pantoea anthophila* CL1, (10) *Pantoea ananatis* PP1, (11) *Fusarium solanum* 3.501, (12) *Fusarium oxysporum f.* sp. *cubense* FOC4, (13) *Aspergillus niger* 3.576, (14) *Colletotrichum gloeosporioides penz.*, (15) *Aspergillus flavus* and (16) *Fusarium nivale*.

To detect whether F9LE has an inhibitory effect on the growth of other phytopathogens, its antibiotic activities were evaluated using 16 kinds of phytopathogenic bacteria or fungi (Supplementary Table S3). *B. amyloliquefaciens* FZB42, as a model bacterium of antimicrobial strains of the genus *Bacillus*, has been successfully used as a biocontrol bacterium in agriculture [11]. FZB42LE was used as a control here. As shown in Figure 5, the lipopeptide extracts obtained from both F9 and FZB42 showed a wide spectrum of antibacterial and antifungal activity. In the antibacterial activity assay, the size of the inhibition zone of F9LE was similar to that of FZB42LE. Both extracts exhibited strongly antibacterial activity to *X. campestris* pv. *campestris* XC1 causing black rot disease, *X. oryzae* pv. *oryzicola* GDIV causing bacterial leaf streak disease and *Ralstonia solanacearum* EP1 causing bacterial wilt disease, while both showed weaker antagonistic action to *Dickeya zeae* EC1 causing rice foot rot disease, *Pectobacterium carotovorum* Er causing bacterial soft rot disease and *Pantoea ananatis* SC7 causing disease symptoms in a wide range of plants. In the antifungal activity assay, F9LE showed stronger antifungal activities than FZB42LE against *Fusarium oxysporum f.* sp. *cubense* causing fusarium wilt of banana, *Fusarium solani* GIM 3.501 causing fusarium wilt of eggplant, *Aspergillus niger* GIM 3.576 causing a disease called ‘black mold’ and *Colletotrichum capsici* causing the leaf spot disease (Figure 5 and Figure S1). However, there was no difference in the bacteriostatic effect on the fungi *Aspergillus flavus* and *Fusarium nivale* (Figure 5). These observations suggest that F9LE has a strong inhibitory effect on the antibiotic activity of certain phytopathogenic fungi.

### 2.8. Identification of the Bioactive Compounds of F9LE by HPLC and LC–ESI–MS

**Figure 6:**
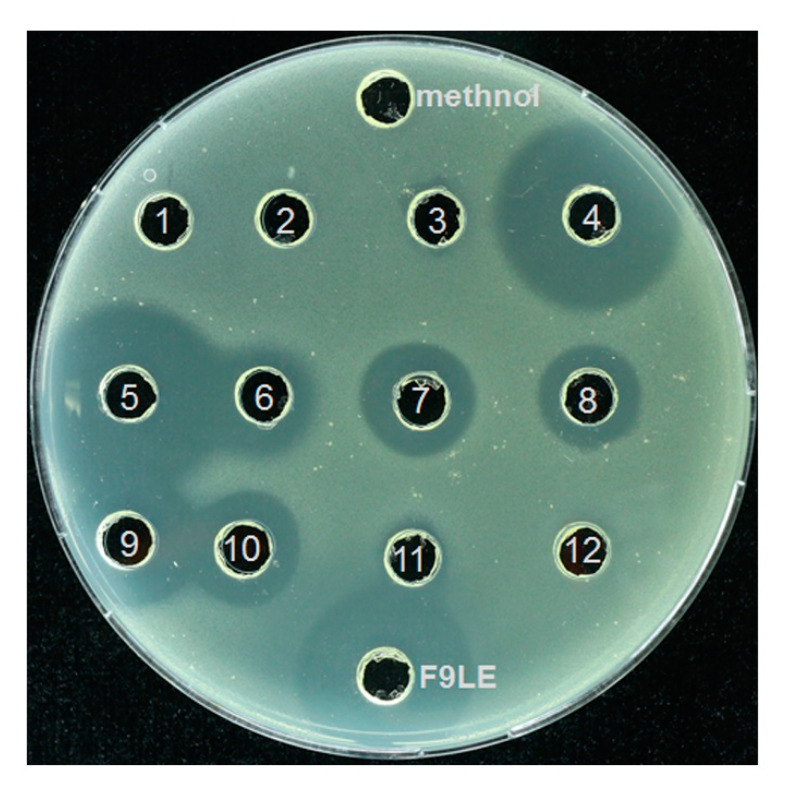
Purification of F9LE by high-performance liquid chromatography. The F9LE obtained by the combination of hydrochloric acid precipitation and acetone extraction was further separated by preparative high-performance liquid chromatography. A total of 12 fractions were collected at regular intervals for elution. The fractions acquired by HPLC were subjected to agar diffusion assay to determine bacteriostatic activity.

The F9LE obtained by the combination of hydrochloric acid precipitation and acetone extraction was further separated by preparative high-performance liquid chromatography (HPLC). A total of 12 fractions were collected at regular intervals for elution. The fractions acquired by HPLC analysis were subjected to agar diffusion assay to determine which fractions have bacteriostatic activity. The results showed that the fractions with an elution time of 20 to 50 min, corresponding to the sample numbers of 4 to 10 (hereafter referred to as fractions 4, 5, 6, 7, 8, 9 and 10), showed strong antagonistic activity on *Xcc* jx-6, as revealed by the surrounding clear inhibition zone (Figure 6). Fractions 4 to 10 were collected and concentrated by rotary evaporation and used for further mass spectrometric analysis.

**Figure 7:**
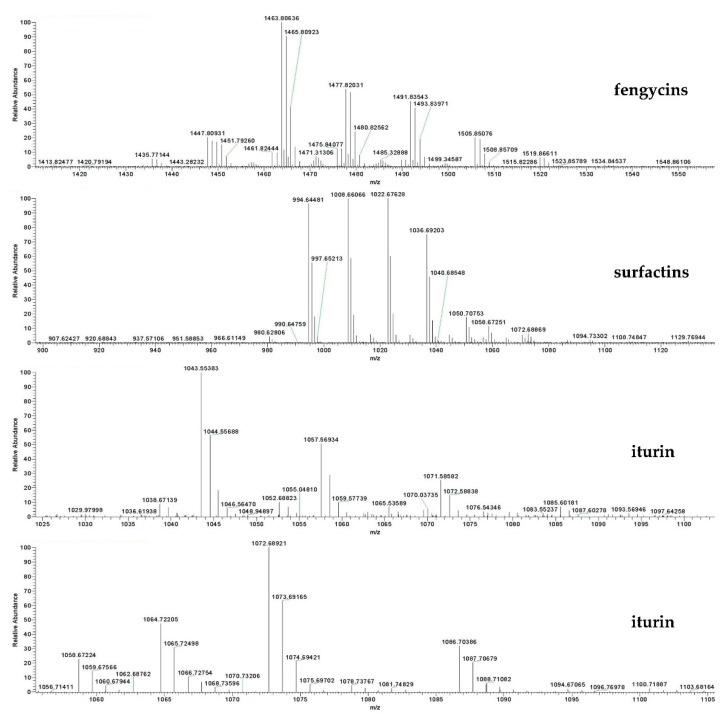
Identification and quantification of lipopeptide compounds from strain F9 and FZB42. Identification of lipopeptide compounds from the acetone extract of strain F9 using HPLC–MS analysis. Mass spectra [M + H]^+^ of LPs are shown. The representative chromatograms of the lipopeptides (iturin, fengycin and surfactin) in acetone extract (3 µL) from strain F9 were used for HPLC–MS analysis.

In order to further explore the molecular masses and element compositions of fractions 4–10, high-performance liquid chromatography and electrospray ionization mass spectrometry (LC–ESI–MS) analysis was performed. Mass spectrometric analysis of fraction 4 revealed a cluster of peaks with *m*/*z* values of 994.6448, 1008.6606, 1022.6762, 1036.6920 and 1050.7075 [M + H]^+^. These peaks differ by 14 Da, suggesting a series of homologous molecules with different lengths of fatty acid chains, belonging to surfactin A/B homologues (Figure 7). The fraction also showed dominant ion peaks at *m*/*z* 1435.7714, 1449.7926, 1463.8063, 1477.8,203, 1491.8354, 1505.8507 and 1519.8661, which confirmed the existence of fengycin A/B homologues (Figure 7). In fractions 5, 6, 7, 8, 9 and 10, the above-mentioned characteristic surfactin and fengycin homologues were also detected. In addition, the analysis of fraction 9 showed that the molecular weights of the iturin A/iturin B homologues were detected as [M + H]^+^ at *m*/*z* = 1043.5538, 1057.5693, 1071.5858, 1085.6018, 1058.6722, 1072.6892, 1086.7038 and 1100.7188, corresponding to C14, C15, C16 and C17 hydroxy fatty acid chains (Figure 7). The various isoforms of each lipopeptide with specific types of homologues detected were summarized and are listed in Table 3. Altogether, three kinds of lipopeptides, including homologues of fengycin A/B, surfactin A/B and iturin A/B, were identified as the main bacteriostatic molecules of F9LE. However, it was interesting that commercial lipopeptides (Sigma-Aldrich), including iturin, fengycin or surfactin or their mixtures, were not active against *Xcc* jx-6 (Supplementary Figure S2). This result indicates that the antibacterial activity of the strain F9 may be caused by the synergistic effect of multiple components, or that there are other components that have an important effect on inhibiting the growth of *Xcc* jx-6. 

## 3. Discussion

Citrus is the crop with the largest cultivation area and highest value of the 15 provinces and cities in southern China. Citrus canker caused by the Gram-negative bacterium *Xcc* infects almost all citrus species [3,4] and poses a serious threat to all citrus-producing areas [2]. Biological control has emerged as a promising strategy that could be applied for the effective management of plant diseases caused by *Xanthomonas* spp. [5,9,10,11,12]. Iturin-like lipopeptides are essential components in the biological control arsenal of *B. subtilis* against the cucurbit pathogenic bacteria *X. campestris* pv. *cucurbitae* [17]. Chitosan isolated from *Euphorbia pulcherrima* markedly inhibits the growth of pathogenic *Xanthomonas* [29]. Difficidin and bacilysin from *B. amyloliquefaciens* FZB42 have antibacterial activities against *X. oryzae* pv. *oryzae* and *X. oryzae* pv. *oryzicola* [18]. The endophytic *B. thuringiensis* strains TbL-22 and TbL-26 were determined to be prospective antagonists against both wild-type and streptomycin-resistant *Xcc* [30]. Reductions in citrus canker severity and incidence were also reported for citrus species treated with the *Bacillus* strains WG6-14 and TKS1-1 [25] and *B. subtilis* (S-12) [26]. Here, we isolated and identified the novel rhizosphere-associated *B. amyloliquefaciens* strain F9, antagonistic against *Xcc* jx-6, from the soil of a citrus farm in southern China. The lipopeptide extract obtained from strain F9 exhibited potent biocontrol activity against *Xcc* jx-6 under both laboratory and greenhouse conditions. Specifically, we discovered that F9LE had a stronger effect on inhibition of the secretion of the extracellular enzymes of *X. citri* jx-6 than FZB42LE did. In addition to its antibacterial activity against *Xcc*, F9LE also exhibited strong bacteriostatic activity against other members of the genus *Xanthomonas*, including *X. campestris* pv. *campestris* strain XC1 and *X. oryzae* pv. *oryzicola* GDIV, in addition to several other pathogenic bacteria. We also presented results showing that F9LE showed stronger antifungal activities than FZB42LE against *Fusarium solanum,* *Fusarium oxysporum*, *Aspergillus niger* and *Colletotrichum* *gloeosporioides penz.* The main antimicrobial compounds in F9LE were identified as three kinds of lipopeptides, including homologues of surfactin, fengycin and iturin. These results indicate that *B. amyloliquefaciens* F9 and its lipopeptide extract may be used as a potential biocontrol agent for combating *Xanthomonas* pathogens and several fungal pathogens.

Species of the bacterial genus *Bacillus* have great agricultural potential due to their ability to produce lipopeptides that have high activity against insects, mites and phytopathogens. These lipopeptides are amphiphilic in nature and, thereby, interfere with biological membrane structures [19,31]. In this study, the effect of the antibacterial lipopeptides of strain F9 on *Xcc* cells was observed using SEM and TEM. Under SEM, bacterial cells treated by F9LE showed shrinkage and deformation, the cell walls of the bacterial cells became thinner, a significantly depressed cavity appeared and the contents leaked. Under TEM, when treated with F9LE, *Xcc* cells shrunk and became deformed, and the cell walls of the bacteria were obviously damaged. There were cavities in the bacterial cytoplasm due to the leakage of the contents. Similar morphological deformities were also observed in *Xcc* treated with ethyl acetate extract of the *B. thuringiensis* strain TbL-22 [30]. In addition, both F9LE and FZB42LE treatments obviously reduced the secretion of the three extracellular enzymes of *Xcc*. In particular, the cellulase activities in F9LE-treated (100 and 150 µg/mL) samples were significantly lower than those in the FZB42LE-treated samples. Similarly, F9LE (25 µg/mL) had stronger inhibitory effects on protease and amylase activities than FZB42LE at the same concentration. These observations suggest that F9LE and FZB42LE may have slightly different lipopeptide compositions, resulting in a stronger inhibitory effect of F9LE than FZB42LE on the extracellular enzyme production of the pathogenic strain *Xcc* jx-6.

To further evaluate the antibacterial ability of the lipopeptide extract of strain F9, we tested the antibacterial activities of commercial lipopeptide standards and compared them with the lipopeptide extract of strain F9. The results showed that all the standard samples of single iturin, fengycin or surfactin or combinations of 2–3 lipopeptides had no antibacterial effect on *Xcc*, indicating that the extracellular metabolite components of strain F9 are complex and its antibacterial activity may be the result of the synergistic effect of multiple components [32]. Alternatively, in strain F9, these three lipopeptides may harbor some modifications that are not found in standard samples and may have an important impact on the antagonistic activity of *Xcc* jx-6. The antimicrobial activity of lipopeptides is generally described as antibacterial activity against fungal phytopathogens, with a few reports addressing their effects on bacteria [9,17,33,34,35,36]. Improvement of the disease prevention effect of strain F9 in vivo and the genetic regulation basis of strain F9 secreting lipopeptides are worthy of further study. These studies will provide a theoretical basis for further elucidating the antibacterial mechanism of strain F9 and the development of bacterial agents. 

## 4. Materials and Methods

### 4.1. Pathogenic Strains and Cultural Conditions 

*Xanthomonas citri* subsp. *citri* strain jx-6 (designated as *Xcc* jx-6) was kindly provided by the Citrus Huanglongbing Research Laboratory of South China Agricultural University. The whole-genome sequence of strain *Xcc* jx-6, isolated from a citrus canker disease-affected tree in Jiangxi Province, China, has been published on NCBI, and the reference sequence is NZ_CP011827.2. Unless otherwise specified, *Xanthomonas* strains were cultured on Luria–Bertani agar (LB; contains 10 g of tryptone, 5 g of yeast extract and 10 g of NaCl per liter) and incubated at 28 °C. *Bacillus* was also cultured on LB agar plates at 28 °C. 

### 4.2. Soil Sample Collection from Citrus Rhizosphere Soil

To isolate the antagonistic strain against *Xcc* jx-6, soil samples were collected from the rhizosphere soil of a healthy citrus orchard in Jiangyong County, Yongzhou City, Hunan Province, China. The temperature of the collection site was 30 °C, and the site is located at 111°3′50” E, 25°5′45” S. The top 5–10 cm layer of rhizosphere soil around the citrus trees was collected. Soil samples were kept in cold storage at 4 °C until processing for the isolation of *Xcc* jx-6 antagonistic strains.

### 4.3. Isolation of Antagonistic Bacteria of Xcc 

A dual culture assay was used here to isolate the antagonistic bacteria of *Xcc* jx-6 as previously described, with modifications [37,38]. The pathogenic strain *Xcc* jx-6 was used as the antagonistic reference strain. Firstly, the bacterial species were recovered from the soil samples as previously described [39]. Each 10 g soil sample was suspended into 90 mL sterile water and cultured at 28 °C with shaking at 180 rpm for 30 min. Then, each 100 μL aliquot of soil culture supernatant was serially diluted at 10×, 100× and 1000×, and the diluted samples were spread on LB agar (1.5% *w*/*v*) containing *Xcc* jx-6 cells (1% *v*/*v*). After these plates were incubated at 28 °C for 48 h, the bacterial colonies that appeared with a clearly transparent circle around them were selected as antagonistic bacteria. Their antagonistic activity on *Xcc* jx-6 was further confirmed by the agar diffusion assay. The selected positive colonies were cultured in LB liquid medium overnight, and 10 µL of cultures was spotted into holes (5 mm in diameter) at the center of the Petri dishes with LB agar (1.5% *w*/*v*) containing *Xcc* jx-6. The same amount of LB medium was spotted as a blank negative control. All these plates were incubated at 28 °C for 48 h, and the diameters of the bacteriostatic zones were measured and recorded. All the potent antagonistic bacteria were stored in 20% glycerol at −80 °C for long-term storage. Then, the strains with the strongest antibacterial activity were selected and the 16S rRNA gene sequence was amplified using the universal primers 27F and 1492R [40]. The obtained PCR products were sequenced, and the nucleotide sequences were compared with the GenBank database using BLAST (https://blast.ncbi.nlm.nih.gov/Blast.cgi on 22 December 2021). 

### 4.4. Characterization of the New Antagonistic Isolate B. amyloliquefaciens F9 

After the strain F9 was grown on LB agar plates at 28 °C for 24 h, the colony morphology was observed with an optical microscope (Leka, Germany). The cell shapes and cellular flagellum type were observed under a Hitachi Hmur7650 transmission electron microscope. The physiological and biochemical tests were carried out according to the bacterial identification program MS(i)/C005-C01 (*Bergey’s Manual of Determinative Bacteriology* (9th Edition), R.E. Buchanan et al., Science Press 2; “Handbook of systematic Identification of Common bacteria” Xiuzhu Dong, Editor-in-Chief, Science Press, 2001) (Table 1). The bacteria with the most potent inhibitory activity were selected and identified according to their 16S rRNA gene sequence, which was amplified using the universal primers 27F and 1492R. The obtained PCR products were purified from agarose gel and entrusted to Guangzhou Aiji Company for DNA sequencing. The partial 16S rDNA nucleotide sequence, 1455 bp in length, was deposited in NCBI GenBank with the accession number MT764967.1. This sequence was compared with entries in the GenBank database using BLAST (https://blast.ncbi.nlm.nih.gov/Blast.cgi, accessed on 12 December 2021). The homologous 16S rDNA sequence was used to construct a phylogenetic tree using MEGA7 software by the neighbor-joining (NJ) algorithm using the Kimura ten-parameter distance.

The homology of the beta-mannanase gene among *Bacillus subtilis* strains can be used to classify and identify *B. subtilis* and *B. amyloliquefaciens* at the intraspecies level [41]. Using primer pairs of Bam-man-1F and Bam-man-1R, a 1275 bp DNA fragment of the β-mannanase gene could be amplified from *B. amyloliquefaciens*. Using primer pairs of Bsu-man-1F and Bsu-man-1R, a 1287 bp DNA fragment could be amplified from the genomic DNA of *B. subtilis* but not *B. amyloliquefaciens.* The primer sequences used are as follows: 

Bam-man-1F: 5′-TCGGTTTCACATCCTTCATC-3′; 

Bam-man-1R: 5′-TTTGTCAGCGTGTCTTCTG-3′; 

Bsu-man-1F: 5′-CAGGCTCACACTTTGTCTTG-3′; 

Bsu-man-1R: 5′-TGAACACAGTCCTGGGTTAG-3′. 

### 4.5. Determination of Antibacterial Activity by Agar Diffusion Assay

The agar diffusion method was used to determine the antibacterial activity as previously described, with modifications [38,42]. Briefly, LB agar plates containing the indicator pathogen *Xcc* were first prepared. The *Xcc* jx-6 cells were grown in LB liquid medium to an OD_600_ of 1.0, and the culture broths were resuspended in LB agar (1.5% *w*/*v*) (cooled to 40 °C) at a ratio of 1:100 and poured into Petri dishes at 15 mL per dish. Holes (5 mm in diameter) were punched in the LB agar plate with a sterile perforator. Then, 10 μL aliquots of the test solution were spotted into 5 mm wells, and the same volume of methanol or PBS buffer was used as a blank control. These plates were incubated at 28 °C for 48 h to allow appearance of a clear transparent inhibition zone. Three replicate groups were set up for each sample. The antibacterial activity was determined by the size (in area) of the bacteriostatic zones on each plate.

### 4.6. Analysis of Antibacterial Stability of Culture Supernatant

The ammonium sulfate precipitation method was used to separate proteins from the cell-free filtrate of strain F9 [35]. The antibacterial activity of the precipitated protein against the pathogen *Xcc* jx-6 was subjected to stability tests. To investigate its temperature stability, 100 µL of the precipitated protein in PBS buffer was incubated for 30 min at temperatures ranging from 25 to 100 °C. To evaluate its stability during enzyme digestion, 100 µL of the precipitated protein solution was incubated with proteinase K at concentrations of 1, 2, 3 and 4 mg/mL at 37 °C for 1 h. The antibacterial properties of the treated samples were evaluated by an agar diffusion assay. Data from both experiments were statistically analyzed using one-way analysis of variance (ANOVA) (*p* < 0.05), and the values are expressed as the means ± standard deviations.

### 4.7. Preparation of Crude Lipopeptide Extract and Its Stability Analysis 

A combination method with acetone extraction after protein precipitation by hydrochloric acid (HCl) was used here to extract crude lipopeptides from the culture supernatant of strain F9 and FZB42 [9,43]. *B. amyloliquefaciens* F9 cells were cultured in the YPD medium at 30 °C for 24 h under agitation (200 rpm). Cell pellets were removed by centrifugation at 8000 rpm for 15 min. The culture supernatant was collected and adjusted to pH 2.0 using 6N HCl and kept at 4 °C overnight. The precipitated protein was further extracted twice with five times the volume of acetone. The resulting acetone extracts of crude lipopeptides were dissolved in methanol or PBS buffer and filtered through a 0.22 μm microfiltration membrane. The protein concentration of the acetone extract was determined using the Protein Quantification Kit (BCA assay, Abbkine) according to the manufacturer’s instructions. The antibacterial activity and stability of lipopeptide extract (resistance to extreme high temperature and proteinase K degradation) were determined by the agar diffusion assay. Data from both experiments were statistically analyzed using one-way analysis of variance (ANOVA) (*p* < 0.05), and the values are expressed as the means ± standard deviations.

### 4.8. Pathogenicity Assays under Greenhouse Conditions

Pathogenicity determination was performed under greenhouse conditions with the infection method of spray inoculation as previously reported, with modifications [44]. The host citrus (Hongjiang, susceptible), 3 years old, was grown in 3 L pots under greenhouse conditions of 26 to 30 °C. Strain *Xcc* jx-6 cells were cultivated in LB at 28 °C and 200 rpm to an optical density (OD) at 600 nm of 1.0. The culture broth was centrifuged at 1073× *g* for 15 min. The pellets were resuspended in PBS buffer and adjusted to an OD_600_ of 1.0. In addition, the lipopeptide extract prepared above was dissolved in PBS buffer for use. The leaves that had expanded by about two-thirds and with almost similar sizes were sterilized with 75% ethanol followed by extensive washing with sterile water. Then, cell suspensions were sprayed on the abaxial surface of the leaves. All test leaves were air-dried and bagged for 24 h, and 0.5 mL lipopeptide extract was then sprayed with a concentration of 244 µg/mL or the same volume of PBS buffer as a nontreatment control. All test leaves were covered with plastic bags, and symptoms of canker spot on leaves began to appear about 10 days post-inoculation. The sprayed leaves were photographed at 30 days post-inoculation, and the leaf canker lesions were recorded. Data from the experiments were statistically analyzed using t-tests (*p* < 0.05), and the values are expressed as the means ± standard deviations. All tests were performed in triplicate.

### 4.9. Determination of Extracellular Enzymes’ Activities

The effect of lipopeptide extract on the extracellular enzyme production of *Xcc*, including amylase, protease and cellulase, was estimated as previously described, with modifications [45]. The lipopeptide extracts of strain F9 and FZB42 were dissolved in methanol at 17.16 and 16.46 mg/mL, respectively, and used as the stock solution. *Xcc* jx-6 cells were cultured in LB medium to an OD_600_ of 1.0. The lipopeptide extracts were diluted to 150, 100, 50 and 25 µg/mL, and *Xcc* cell suspensions with different concentrations of lipopeptide extract were then used for the determination of extracellular enzyme activity. All the culture plates were incubated at 28 °C for 3 days.

For the amylase activity assay, the same amount (2 µL) of *Xcc* cell suspensions with different concentrations of lipopeptide extract was spotted onto NYG agar plates (5 g of peptone, 3 g of yeast extract and 20 g of glycerol per liter) containing 1% (*w*/*v*) starch. After incubation for 3 d at 28 °C, the plates were flooded with 1% iodine in 2% potassium iodide. The amylase activity was measured based on the zone area of clearance around the blue background. For the protease activity assay, 2 µL of *Xcc* cell suspensions with different concentrations of lipopeptide extract was spotted onto NYG agar plates containing 1% (*w*/*v*) nonfat milk powder. Protease activity was measured by the zone area of the transparent hydrolysis circle produced. For the cellulase activity assay, 2 µL *Xcc* cell suspensions with different concentrations of lipopeptide extract were spotted onto NYG agar plates containing 1% (*w*/*v)* hydroxymethyl ethyl cellulose. After incubation for 3 days, the plates were stained with 0.1% Congo red solution for 0.5 h and then decolorized with 1 M NaCl until a clear yellow zone around the colony was observed. Cellulase enzyme activity was measured based on the yellow zone area. Data from these experiments were statistically analyzed using one-way analysis of variance (ANOVA) (*p* < 0.05), and the values are expressed as the means ± standard deviations.

### 4.10. Scanning Electron Microscopy (SEM) and Transmission Electron Microscopy (TEM) Studies

SEM and TEM assays were used to observe the effect of antibacterial lipopeptides on *Xcc* jx-6 cells at the ultrastructural level with a slightly modified version of the procedure described in [18,30]. *Xcc* cells were cultured to the logarithmic phase of growth at 28 °C and 150 rpm. Aliquots of 0.5 mL of cell cultures were diluted into 5 mL liquid LB containing 200 µg/mL F9LE (lipopeptide-treated sample) or into 5 mL LB medium (nontreatment sample). The resulting solutions were cultured at 28 °C and 150 rpm for 12 h. SEM specimens and TEM specimens were prepared as follows.

For SEM analysis, *Xcc* jx-6 cells were collected and washed 3 times with 100 mM phosphate buffer solution of pH 7.2. The cells were fixed with glutaraldehyde (2.5%) for 4 h, washed 3 times with 100 mM phosphate buffer and then post-fixed in 1% osmium tetroxide for 30 min. Dehydration was performed sequentially with 50, 70, 80, 90 and 100% ethanol with each step for 15 min. The dehydrated specimen was placed in a high-vacuum evaporator, coated with gold particles and observed under a Zeiss EVO MA 15 Scanning Electron Microscope (Berlin, Germany). For TEM analysis, *Xcc* jx-6 cells were fixed with glutaraldehyde (2.5%) for 4 h and washed three times with 100 mM phosphate buffer of pH 7.2. Dehydration was performed sequentially with 70, 80, 90, 95 and 100% ethanol for 15 min at each step. The samples were embedded in resin, polymerized at 70 °C for 9 h, sliced with an ultra-thin slicing machine and put on a 400-mesh uncoated copper wire; then, they were dyed with uranyl acetate for 15 min and counter-stained with lead citrate for 15 min, washed with sterile distilled water, dried at 37 °C and observed under the Thermo Scientific Talos L120C TEM.

### 4.11. Determination of Antimicrobial Spectrum of F9LE 

The pathogenic microorganisms tested in this study are listed in Supplementary Table S3. The F9 crude lipopeptide extract was dissolved in methanol to 17.46 mg/mL (*w*/*v*) and used as the stock. The antimicrobial activity of F9LE and FZB42LE against 10 kinds of pathogenic bacteria was tested using the agar diffusion method. The 10 pathogenic bacteria, shown in Supplementary Table S3, were cultured in LB medium. Briefly, 10 µL of lipopeptide extract was pipetted into the wells of the test plate, and the same amount of methanol was used as a control. The plates were incubated at 28 °C for 36 h, and the formation of clear zones around the holes was used as an indicator of antibacterial activity. Additionally, the bacteriostatic activity of F9LE against 6 kinds of pathogenic fungi was tested by the plate confrontation method as previously described, with minor modifications [36]. The 6 kinds of pathogenic fungi, shown in Supplementary Table S3, were activated on potato dextrose agar (PDA) plates and cultured to grow hypha at 28 °C. A fresh mycelium cake of the investigated fungi (5 mm in diameter) was placed at the center of the PDA (1.5% *w*/*v)* in Petri dishes (9 cm diameter). Four holes were drilled with a 5 mm sterile perforator around the fungi cakes, and 10 µL of crude lipopeptide extract was added into the wells of the test plate and cultured at 28 °C.

### 4.12. HPLC Purification of Putative Bioactive Compounds 

After detection of the antimicrobial activity of the crude lipopeptides by the agar diffusion method, the lipopeptide extract was further purified by preparative high-performance liquid chromatography (HPLC) (Agilent 1260 Infinity II Preparative LC System). The chromatographic column was a C18 column (5 µm, 10 mm × 250 mm). The effluent was monitored at UV (200–400 nm) wavelength. The solvent gradient profile used buffer A (mobile phase: 0.1% (*v*/*v*) aqueous formic acid) and buffer B (elution phase: acetonitrile) at a flow rate of 3 mL/min. Sample elution started with 40% buffer B, followed by a linear gradient increasing to 100% buffer B over 60 min. The fractions of the eluate were collected at regular intervals with 5 min per fraction, and a total of 12 components were obtained. The antibacterial activities of each fraction were determined by agar diffusion assay as described above. The fractions with antimicrobial activity were concentrated and evaporated. Subsequently, the chemical constituents of the antimicrobial substances were determined by liquid chromatography–mass spectrometry (LC–MS). 

### 4.13. LC–ESI–MS Analysis of Bioactive Compounds of F9LE 

Chemical constituents of antimicrobial F9LE were determined by LC–ESI–MS analysis. The Thermo Scientific Q Exactive Focus Orbitrap LC–MS/MS System (Waltham, MA, USA) was used for identification of the bioactive molecular species from F9LE. All analyses of liquid chromatography were performed using the Ultimate^TM^ 3000 UPLC system (Thermo Fisher Scientific, Waltham, MA, USA) equipped with a Waters ACQUITY UPLC HSST3 column (1.8 µm, 2.1 mm × 100 mm) and monitored at 210 nm. A gradient was achieved by changing the ratio of the following two eluents: eluent A, water; eluent B, acetonitrile. Sample elution started with 80% buffer B over 20 min at a flow rate of 0.3 mL/min. The separated compounds were directly electrosprayed into the mass spectrometer using the electrospray ionization (ESI) source conditions set as follows: ion spray voltage at 3.5 kV, sheath gas at 40 units and capillary temperature at 320 °C. The data were collected in positive ionization mode with dependent MS acquisition in the range of *m/z* 850–2000. The full-scan spectra were collected at a resolution of 70,000. 

## Figures and Tables

**Table 1:** The biochemical and physiological characteristics of strain F9.

Characteristic	Result	Characteristic	Result
Catalase	+	Phenylalanine deaminase	−
Oxidase	−	2% Salt tolerance test with 2% NaCl	+
Gelatin hydrolysis	+	Salt tolerance test with 5% NaCl	+
V-P determination	+	Salt tolerance test with 7% NaCl	+
Propionate	−	Salt tolerance test with 10% NaCl	−
Urease	−	Amylolysis	+
Anaerobic culture	+	Glucose to acid	+
Nitrate reduction	+	Citrate	+
Lecithin enzyme	−	Indole test	−
Tyrosine hydrolysis	−	Casein hydrolysis	+
Broth at pH 5.7	+	Broth at pH 6.8	+

Note: + denotes positive, − denotes negative.

**Table 2:** Biochemical characterization of the antibacterial activity of strain F9 lipopeptide extract.

Treatment Conditions	Diameter of Inhibition Zones (mm)	Relative Percentage of Control (%)
Temperature (℃)		
25.0 (control)	20.00 ± 1.47	100.0
45.9	19.83 ± 1.25	99.2
70.2	19.83 ± 0.62	99.2
81.6	16.50 ± 0.71	82.5
85.5	16.33 ± 0.85	81.7
91.0	16.17 ± 1.03	80.9
95.4	14.17 ± 1.25 *	70.9
100.0	13.50 ± 1.87 *	67.5
Proteinase K (mg/mL)		
0.0 (control)	14.33 ± 0.24	100.0
1.0	12.50 ± 0.71	87.2
2.0	12.50 ± 1.08	87.2
3.0	13.00 ± 1.08	90.7
4.0	13.17 ± 0.94	91.9

* represents significant differences (*p* < 0.05).

**Table 3:** Primary peaks detected by LC–MS analysis of the lipopeptides produced by strain F9.

Lipopeptide Isoforms	Fatty Acid Chain	Calculated (*m/z*)		
		[M + H]^+^	[M + Na]^+^	[M + K]^+^
Iturin A/Mycosubtilin	C14	1043.5538	1065.5538	1081.5538
	C15	1057.5693	1079.5693	1095.5693
	C16	1071.5858	1093.5858	1109.5858
	C17	1085. 6018	1107.6018	1123.6018
Iturin B	C15	1058.6722	1080.6722	1096.6722
	C16	1072.6892	1094.6892	1110.6892
	C17	1086.7038	1108.7038	1127.7038
	C18	1100.7188	1122.7188	1138.7188
Surfactin	C13	994.6448	1016.6448	1032.6448
	C13, C14	1008.6606	1030.6606	1046.6606
	C14, C15	1022.6762	1044.6762	1060.6762
	C15, C16	1036.6920	1058.6920	1074.6920
	C16	1050.7075	1072.7075	1088.7075
Fengycin	C14	1435.7714	1457.7714	1473.7714
	C15	1449.7926	1471.7926	1487.7926
	C16, C14	1463.8063	1485.8063	1501.8063
	C17, C15	1477.8203	1499.8203	1515.8203
	C18, C16	1491.8354	1513.8354	1529.8354
	C17	1505.8507	1527.8507	1543.8507
	C18	1519.8661	1541.8661	1557.8661

## Data Availability

Not applicable.

## Supplementary Materials

The following supporting information can be downloaded at: https://www.mdpi.com/article/10.3390/plants11030457/s1, Figure S1. Demonstration of the inhibition effects of F9LE agaist 4 fungal pathogenic strain. (A) Photographs of antimicrobial activities of crude lipopeptides against 4 pathogenic fungi were listed. *Fusarium oxysporum f*. sp. Cubensis FOC4 causing fusarium wilt of banana, Fusarium solani GIM 3.501 causing fusarium wilt of eggplant, Colletotrichum capsici causeing the leaf spot disease, and Aspergillus niger GIM 3.576 causing a disease called ‘black mold’. (B) The effect of crude lipopeptides against 4 pytopathogenic fungi was qualitatively determined by area of the inhibition zone. *B. amyloliquefaciens* FZB42 was used as a control. Figure S2. The antibacterial ability of strain F9 crude lipopeptide extract and the lipopeptide standards. The antibacterial activity of commercial lipopeptide standards and the lipopeptide extract of strain F9 was test by a gar diffusion assay. Left panel, single lipopeptide iturin, fengycin, and surfactin was tested; right panel, 2 or 3 lipopeptide mixtures were tested. Table S1. 22 isolates were screened and identified as having antibacterial activity against strain Xcc jx-6. These strains displayed inhibition zones ranging from 10 to 30 mm in diameter in a agar diffusion assay. Table S2. Biochemical characterization of the antibacterial activity of the precipitated extract of strain F9 by ammonium sulfate. For the temperature sensitivity test, the precipitated extract of strain F9 was treated at 25 °C, 45.9 °C, 70.2 °C, and till to 100 °C for 30 min. The effect of proteinase K was test by treated the precipitated extract of strain F9 with 1 mg/mL, 2 mg/mL, 3 mg/mL and 4 mg/mL of enzyme at 37 °C for 1 h. After the treatments, the activity of culture filtrate of strain F9 was tested by the agar diffusion assay. Table S3. Pytopathogenic microorganisms tested in antimicrobial spectrum analysis of lipopeptide extract obtained from srain F9.
