# Supplementary material for: Isolation of the Novel Strain Bacillus amyloliquefaciens F9 and Identification of Lipopeptide Extract Components Responsible for Activity against Xanthomonas citri subsp. citri
Source: Plants (Basel). 2022 Feb 7;11(3):457. doi: 10.3390/plants11030457 (PMC8840523; doi:10.3390/plants11030457)
Supplement: Supplementary file 1 [file plants-11-00457-s001.zip › plants-1558810-supplementary.pdf]

Supplemental material

Isolation of the novel strain *Bacillus amyloliquefaciens* F9 and identification of lipopeptide extract components responsible for activity against *Xanthomonas citri* subsp. *citri*

Xin Wang [1,#], Liqiong Liang [1,#], Hang Shao [1], Xiaoxin Ye [1], Xiaobei Yang [1], Xiaoyun Chen [1], Yu Shi [1], Lianhui Zhang [1,2], Linghui Xu [1,2*], Junxia Wang [1,2*]

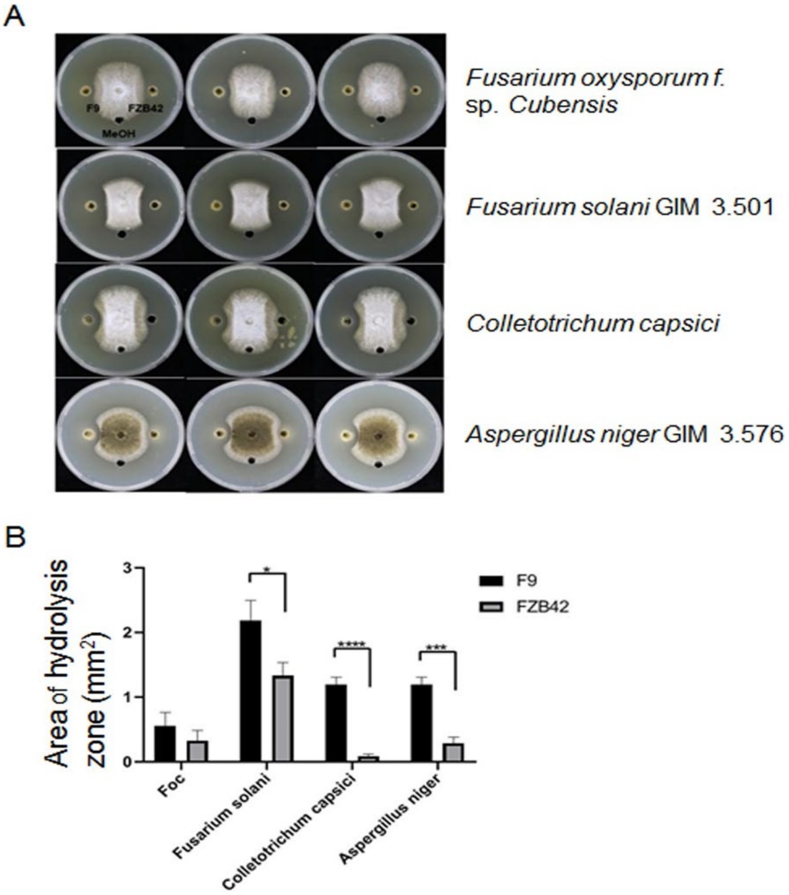

Supplementary Figure S1. Demonstration of the inhibitory effects of F9LE against 4 fungal pathogenic strain. (A) Photographs of antimicrobial activities of crude lipopeptides against 4 pathogenic fungi were listed. *Fusarium oxysporum f. sp.*

cubense FOC4 causing fusarium wilt of banana*, Fusarium solani* GIM 3.501 causing fusarium wilt of eggplant, *Colletotrichum capsici* causeing the leaf spot disease, and *Aspergillus niger* GIM 3.576 causing a disease called ‘black mold’. (B) The effect of crude lipopeptides against 4 pytopathogenic fungi was qualitatively determined by area of the inhibition zone. *B. amyloliquefaciens* FZB42 was used as a control.

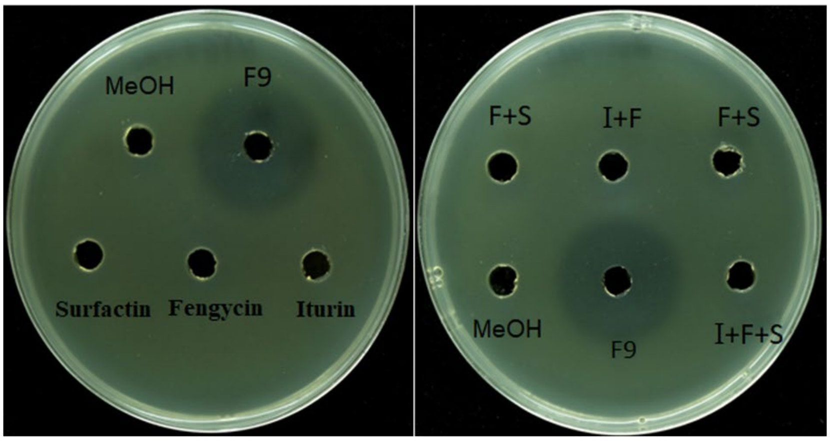

Supplementary Figure S2. The antibacterial ability of strain F9 crude lipopeptide extract and the lipopeptide standards. The antibacterial activity of commercial lipopeptide standards and the lipopeptide extract of strain F9 was test by an agar diffusion assay. Left panel, single lipopeptide iturin, fengycin, and surfactin was tested; right panel, 2 or 3 lipopeptide mixtures were tested.

Supplementary Table S1. 22 isolates with antibacterial activity against *Xcc* JX-6 strain were screened out

Strains	Diameter of Inhibition zone （mm）	Strains	Diameter of Inhibition zone （mm）
F9	30.2 ± 0.33	C4	18.2 ± 0.92
H7	29.6 ± 0.24	E5	17.9 ± 0.21
F6	28.8 ± 0.48	D3	17.8 ± 0.64
F4	23.8 ± 0.12	E9	17.5 ± 0.62
E7	23.1 ± 0.68	E7-1	16.4 ± 0.88
HG-7	22.7 ± 0.31	E8-1	15.3 ± 0.50
B6	22.6 ± 0.05	HB-7	15.2 ± 0.54
E3	22.6 ± 0.21	E6	14.0 ± 0.81
B1	22.4 ± 0.29	D5	13.3 ± 0.37
B12	22.2 ± 0.26	D6	12.1 ± 0.29
E10	21.8 ± 0.25	HD-3	9.7 ± 0.17

Supplementary Table S2. Biochemical characterization of the antibacterial activity of the precipitated extract of strain F9 by ammonium sulfate

Treatment conditions	Diameter of Inhibition zones（mm）	Relative percentage of control (%)
Temperature (℃)		
25.0 (contol)	23.50 ± 1.08	100.0
45.9	22.83 ± 1.55	97.1
70.2	22.50 ± 1.50	95.7
81.6	19.67 ± 0.24*	83.7
85.5	19.50 ± 1.08*	83.0
91.0	19.33 ± 0.62*	82.3
95.4	18.17 ± 0.47**	77.3
100.0	17.80 ± 0.85***	75.7
Proteinase K (mg/ml）		
0.0 (control)	25.00 ± 1.63	100.0
1.0	24.67 ± 1.25	98.7
2.0	25.33 ± 1.70	101.3
3.0	25.33 ± 1.70	101.3
4.0	24.67 ± 1.25	98.7

Asterisks indicate significant differences (*$p < 0.05$, **$p < 0.01$， ***$p < 0.001$)

Supplementary Table S3. Pytopathogenic microorganisms tested in antimicrobial spectrum analysis of crude lipopeptides obtained from srain F9

Pathogenic microorganisms	Source or reference
Xanthomonas citri pv. citri strain jx-6	Lab storage
Xanthomonas campestris pv.campestris strain XC1	Chunyan Zhang, Mingfa
Xanthomonas oryzae pv. oryzicola GDIV	Liexian Zeng, Congying Wang
Ralstonia solanacearum EP1	Peng Li, Dechen Wang,
Burkholderia cenocepacia H111	Chaoyu Cui, Chunxi Yang,
Dickeya zeae EC1	Ming Hu, Jieling Li,
Pectobacterium carotovorum Er	Jieling Li, Ming Hu
Pantoea ananatis SC7	Jieling Li, Ming Hu
Pantoea anthophila CL1	Jieling Li, Ming Hu
Pantoea ananatis PP1	Jieling Li, Ming Hu
Fusarium solani GIM 3.501	Guangdong Microbial Strain Preservation Center
Fusarium oxysporum f. sp. cubense FOC4	Min-Hui Li, Xiao-Ling Xie
Aspergillus niger GIM 3.576	Guangdong Microbial Strain Preservation Center
Colletotrichum gloeosporioides penz.	Jieling Li, Ming Hu
Aspergillus flavus GIM 3.493	Guangdong Microbial Strain Preservation Center
Fusarium nivale GIM 3.503	Guangdong Microbial Strain Preservation Center

1. **Chunyan Zhang, Mingfa Lv,** Wenfang Yin, Tingyan Dong, Changqing Chang, Yansong Miao, Yantao Jia, and Yinyue Deng, Xanthomonas campestris Promotes Diffusible Signal Factor Biosynthesis and Pathogenicity by Utilizing Glucose and Sucrose from Host Plants. Mol Plant Microbe Interact., 2019, 32(2):157-166. doi: 10.1094/MPMI-07-18-0187-R.

2. **Peng Li, Dechen Wang**, Jinli Yan, Jianuan Zhou, Yinyue Deng, Zide Jiang, Bihao Cao, Zifu He, and Lianhui Zhang. Genomic Analysis of Phylotype Strain EP1 Reveals Substantial Divergence from Other Strains in the Ralstonia solanacearum Species Complex. Frontiers in Microbiology, 2016, 7, 1719

3. **Chaoyu Cui, Chunxi Yang**, Shihao Song, Shuna Fu, Xiuyun Sun Liang Yang,5 Fei He,3 Lian-Hui Zhang, Yongliang Zhang and Yinyue Deng. A novel two-component system modulates quorum sensing and pathogenicity in Burkholderia cenocepacia. Molecular Microbiology, 2018, 108 (1), 32 – 44

4. **Liexian Zeng, Congying Wang**, Aiqing Feng, Chen Shen, Jing Su, Wen-juan Wang, Shengyuan Wu, Jianyuan Yang, Xiaoyuan Zhu. Resistance analysis on major resistant genes of rice bacterial blight in different donors and genetic background. Acta Phytopathologica Sinica, 2016, 46 (4): 514-520

5. **Ming Hu, Jieling Li,** Ruiting Chen, Wenjun Li, Luwen Feng, Lei Shi, Yang Xue, Xiaoyin Feng, Lianhui Zhang and Jianuan Zhou., BMC Microbiology (2018) 18:136. Dickeya zeae strains isolated from rice, banana and clivia rot plants show great virulence differentials. BMC Microbiology, 2018, 18:136.

6. **Jieling Li, Ming Hu**, Yang Xue, Xia Chen, Guangtao Lu, Lianhui Zhang, Jianuan Zhou, Screening, Identification and Efficacy Evaluation of Antagonistic Bacteria for Biocontrol of Soft Rot Disease Caused by Dickeya zeae. Microorganisms, 2020, 9; 8(5): 697.

7. **Min-Hui Li, Xiao-Ling Xie**, Xian-Feng Lin, Jin-Xiu Shi, Zhao-Jian Ding, Jin-Feng Ling, Ping-Gen Xi, Jia-Nuan Zhou, Yue-Qiang Leng, Shao-Bin Zhong, Zi-De Jiang. Functional characterization of the gene FoOCH1 encoding a putative a-1,6-mannosyltransferase in Fusarium oxysporum f. sp. Cubense. Fungal Genetics and Biology, 2014, 65, 1 – 13.
